# Validation of the revised 2018 AAST-OIS classification and the CT severity index for prediction of operative management and survival in patients with blunt spleen and liver injuries

**DOI:** 10.1007/s00330-020-07061-8

**Published:** 2020-07-21

**Authors:** Dagmar Morell-Hofert, Florian Primavesi, Margot Fodor, Eva Gassner, Veronika Kranebitter, Eva Braunwarth, Matthias Haselbacher, Ulrich Peter Nitsche, Stefan Schmid, Michael Blauth, Dietmar Öfner, Stefan Stättner

**Affiliations:** 1 Department of Radiology, Medical University of Innsbruck, Anichstrasse 35, 6020 Innsbruck, Austria; 2 Department of Visceral, Transplant and Thoracic Surgery, Centre of Operative Medicine, Medical University of Innsbruck, Anichstrasse 35, 6020 Innsbruck, Austria; 3 Department of Trauma Surgery, Centre of Operative Medicine, Medical University of Innsbruck, Anichstrasse 35, 6020 Innsbruck, Austria; 4 Department of Surgery, Klinikum rechts der Isar, Technical University of Munich, Ismaningerstrasse 22, 81675 Munich, Germany; 5 Department of General and Surgical Intensive Care Medicine, Medical University of Innsbruck, Anichstrasse 35, 6020 Innsbruck, Austria; 6 Department of General, Visceral- and Vascular Surgery, Salzkammergut Klinikum, Dr.-Wilhelm-Bock-Straße 1, 4840 Vöcklabruck, Austria

**Keywords:** Splenic rupture, Blunt injuries, Trauma severity indices, Diagnostic imaging, Mortality

## Abstract

**Objectives:**

Non-operative management (NOM) is increasingly utilised in blunt abdominal trauma. The 1994 American Association of Surgery of Trauma grading (1994-AAST) is applied for clinical decision-making in many institutions. Recently, classifications incorporating contrast extravasation such as the CT severity index (CTSI) and 2018 update of the liver and spleen AAST were proposed to predict outcome and guide treatment, but validation is pending.

**Methods:**

CT images of patients admitted 2000–2016 with blunt splenic and hepatic injury were systematically re-evaluated for 1994/2018-AAST and CTSI grading. Diagnostic accuracy, diagnostic odds ratio (DOR), and positive and negative predictive values were calculated for prediction of in-hospital mortality. Correlation with treatment strategy was assessed by Cramer V statistics.

**Results:**

Seven hundred and three patients were analysed, 271 with splenic, 352 with hepatic and 80 with hepatosplenic injury. Primary NOM was applied in 83% of patients; mortality was 4.8%. Comparing prediction of mortality in mild and severe splenic injuries, the CTSI (3.1% vs. 10.3%; diagnostic accuracy = 75.4%; DOR = 3.66; *p* = 0.006) and 1994-AAST (3.3% vs. 10.5%; diagnostic accuracy = 77.9%; DOR = 3.45; *p* = 0.010) were more accurate compared with the 2018-AAST (3.4% vs. 8%; diagnostic accuracy = 68.2%; DOR = 2.50; *p* = 0.059). In hepatic injuries, the CTSI was superior to both AAST classifications in terms of diagnostic accuracy (88.7% vs. 77.1% and 77.3%, respectively). CTSI and 2018-AAST correlated better with the need for surgery in severe vs. mild hepatic (Cramer V = 0.464 and 0.498) and splenic injuries (Cramer V = 0.273 and 0.293) compared with 1994-AAST (Cramer V = 0.389 and 0.255; all *p* < 0.001).

**Conclusions:**

The 2018-AAST and CTSI are superior to the 1994-AAST in correlation with operative treatment in splenic and hepatic trauma. The CTSI outperforms the 2018-AAST in mortality prediction.

**Key Points:**

*• Non-operative management of blunt abdominal trauma is increasingly applied and correct patient stratification is crucial.*

*• CT-based scoring systems are used to assess injury severity and guide clinical decision-making, whereby the 1994 version of the American Association of Surgery of Trauma Organ Injury Scale (AAST-OIS) is currently most commonly utilised.*

*• Including contrast media extravasation in CT-based grading improves management and outcome prediction. While the 2018-AAST classification and the CT-severity-index (CTSI) better correlate with need for surgery compared to the 1994-AAST, the CTSI is superior in outcome-prediction to the 2018-AAST.*

## Introduction

Advances in imaging techniques have led to the development of multiple radiological classification systems for blunt splenic and hepatic injuries [1, 2]. These are used as primary screening tools in early decision-making (operative [OM] vs. non-operative [NOM] management) [3]. The refinement of CT scanning is partially responsible for the increasing tendency towards NOM in hemodynamically stable patients [4–8]. Despite their widespread use, only few of these classifications have been adequately validated [9] and several studies have proven CT findings inaccurate to determine management and outcome [1, 10–12].

Currently, the most widely accepted scoring system is the revised Organ Injury Scale (OIS) of the American Association for Surgery of Trauma (AAST; Table 1) [13, 14]. Since its initial publication in 1989, with first revision in 1994, it has been regarded as the gold standard to classify traumas [1]. Despite frequent clinical use, the purpose of this classification initially was to provide an anatomical description, rather than to guide clinical pathways [1, 13, 14]. Low-grade AAST-OIS lesions (I–III) are often considered non-severe and treated with NOM, whereas high-grade lesions (>III) tend to lead towards surgery. However, in a number of cases, hemodynamically stable major injury patients can be successfully treated non-operatively [15–18], while minor-grade lesions with hemodynamic instability require OM. Therefore, in determining the treatment strategy, the AAST-OIS should always be supplemented by hemodynamic status and associated injuries.

**Table 1:** Organ injury scale (OIS) of the American Association for Surgery of Trauma (AAST): 1994 revision

Spleen			Liver	
Grade	Type	Injury description	Type	Injury description
I	Haematoma	Subcapsular, < 10% surface area	Haematoma	Subcapsular, < 10% surface area
	Laceration	Capsular tear, < 1% parenchymal depth	Laceration	Capsular tear, < 1% parenchymal depth
II	Haematoma	Subcapsular, 10–50% surface area	Haematoma	Subcapsular, 10–50% surface area
		Intra-parenchymal, < 5 cm in diameter		Intra-parenchymal, < 5 cm in diameter
	Laceration	Capsular tear, 1–3 cm parenchymal depth that does not involve a trabecular vessel	Laceration	Capsular tear, 1–3 cm parenchymal depth that does not involve a trabecular vessel
III	Haematoma	Subcapsular, > 50% surface area or expanding	Haematoma	Subcapsular, > 50% surface area or expanding
		Ruptured subcapsular or parenchymal haematoma		Ruptured subcapsular or parenchymal haematoma
		Intra-parenchymal haematoma, ≥ 5 cm or expanding		Intra-parenchymal haematoma, ≥ 10 cm or expanding
	Laceration	> 3 cm parenchymal depth or involving trabecular vessels	Laceration	> 3 cm parenchymal depth
IV	Laceration	Laceration involving segmental or hilar vessels producing major devascularisation (> 25% of spleen)	Laceration	Parenchymal disruption involving 25–75% of hepatic lobe or 1–3 Couinaud’s segments within the single lobe.
V	Laceration	Complete shattered spleen	Laceration	Parenchymal disruption involving > 75% of hepatic lobe or > 3 Couinaud’s segments within the single lobe.
	Vascular	Hilar vascular injury which devascularises spleen	Vascular	Juxtavenous hepatic injuries; i.e. retrohepatic vena cava/central major hepatic veins
VI			Vascular	Hepatic Avulsion
Additional points:				
Advance one grade for multiple injuries up to grade III				

The 1994-AAST revision does not include contrast media extravasation or arterial pseudo-aneurysms, which studies have shown to be a major factor for NOM failure [19, 20]. Hence, a novel CT-based classification system for splenic injuries was presented in 2007 by Marmery et al [21]. This CT severity index (CTSI) classifies splenic lesions into four grades (I–IV) (Table 2), whereby non-bleeding splenic vascular injuries, including pseudo-aneurysm and arteriovenous fistula, and intraparenchymal/intraperitoneal extravasation constitute high-severity (grade IV) criteria. Compared with the AAST-1994 classification, the CTSI proved to better predict if patients needed splenic artery embolization or OM [21–23]. However, an adaption for liver injuries has so far not been provided. In 2018, the AAST published an update for their classification of spleen and liver injuries implementing imaging features of contrast media extravasation to improve its value for clinical patient management (Table 3) [24]. Both the CTSI and 2018-AAST classifications have so far not been independently validated.

**Table 2:** CT severity index (CTSI) for spleen and liver injury (AAST): CTSI

Spleen		Liver	
Grade	Injury description	Grade	Injury description
I	Subcapsular haematoma < 1 cm depth	I	Subcapsular haematoma < 1 cm depth
	Laceration < 1 cm depth		Laceration < 1 cm depth
	Parenchymal haematoma < 1 cm diameter		Parenchymal haematoma < 1 cm diameter
II	Subcapsular haematoma 1–3 cm depth	II	Subcapsular haematoma 1–5 cm depth
	Laceration 1–3 cm parenchymal depth		laceration 1–5 cm depth
	Parenchymal haematoma 1–3 cm diameter		Parenchymal haematoma 1–5 cm diameter
III	Laceration > 3 cm depth	III	Laceration > 5 cm depth
	Parenchymal haematoma > 3 cm diameter		Parenchymal haematoma > 5 cm diameter
			Subcapsular haematoma > 5 cm depth
IVA	Active intraparenchymal and subcapsular splenic bleeding	IVA	Active intraparenchymal and subcapsular splenic bleeding
	Splenic vascular injury (pseudoaneurysm or AV-fistula)		Hepatic vascular injury (pseudoaneurysm or AV-fistula)
	Shattered spleen		Shattered liver
IVB	Active intraperitoneal bleeding	IVB	Active intraperitoneal bleeding

**Table 3:** Organ injury scale (OIS) of the American Association for Surgery of Trauma (AAST): 2018 revision

Spleen			Liver	
Grade	Type	Injury description	Type	Injury description
I	Haematoma	Subcapsular, < 10% surface area	Haematoma	Subcapsular, < 10% surface area
	Laceration	Capsular tear, < 1% parenchymal depth	Laceration	Capsular tear, < 1% parenchymal depth
II	Haematoma	Subcapsular, 10–50% surface area	Haematoma	Subcapsular, 10–50% surface area
		Intra-parenchymal, < 5 cm in diameter		Intra-parenchymal, < 10 cm in diameter
	Laceration	1–3 cm parenchymal depth	Laceration	Capsular tear, 1–3 cm parenchymal depth, < 10 cm length
III	Haematoma	Subcapsular, > 50% surface area	Haematoma	Subcapsular, > 50% surface area of ruptured subcapsular or parenchymal haematoma
		Ruptured subcapsular or parenchymal haematoma ≥5 cm		intraparenchymal > 10 cm
	Laceration	> 3 cm parenchymal depth or involving trabecular vessels	Laceration	Capsular tear, > 3 cm parenchymal depth
			Vascular	Vascular injury with active bleeding contained within liver parenchyma
IV	Laceration	Parenchymal laceration involving segmental or hilar vessels producing > 25% devascularisation	Laceration	Parenchymal disruption involving 25–75% hepatic lobe or involves 1–3 Couinaud segments
	Vascular	Any injury in the presence of a splenic vascular injury or active bleeding confined within splenic capsule	Vascular	Vascular injury with active bleeding breaching the liver parenchyma into the peritoneum
V	Laceration	Shattered spleen	Laceration	Parenchymal disruption involving > 75% of hepatic lobe
	Vascular	Any injury in the presence of splenic vascular injury with active bleeding extending beyond the spleen into the peritoneum	Vascular	Juxtavenous hepatic injuries; i.e. retrohepatic vena cava/central major hepatic veins
Additional points:				
Advance one grade for multiple injuries up to grade III				
*Vascular injury* (i.e. pseudoaneurysm or AV fistula), appears as a focal collection of vascular contrast which decreases in attenuation on delayed images				
*Active bleeding*, focal or diffuse collection of vascular contrast which increases in size or attenuation on a delayed phase				

This study therefore aims to evaluate an adapted hepatic version of the CTSI regarding its predictive value in terms of patient outcome and correlation with the need for operative management. Furthermore, we compare and validate the CTSI, 2018-AAST and 1994-AAST classifications in blunt hepatic and splenic injuries to determine their value in clinical management.

## Materials and methods

All patients with blunt splenic or hepatic injuries admitted to our hospital between 2000 and 2016 were retrospectively evaluated. The study conforms to the STROBE guidelines [25] and was approved by the institutional ethics board (protocol-number EK1034/2017), waiving the need for informed consent.

In our centre, the vast majority of polytrauma patients receive contrast-enhanced, whole-body multidetector spiral CT (MDCT) on admission. Patients in which only sonographic or non-contrast-enhanced CT assessment had been performed, e.g. due to contraindication for contrast media administration, were excluded from the analysis. Primary MDCT-based trauma evaluation is based on a designated protocol including portal venous phase abdominal imaging. In the case of suspected vascular lesion, additional image acquisition in arterial or delayed phase follows. When patients had received recent external MDCT according to these standards before referral to our centre, usually imaging was not repeated on admission.

MDCTs were re-evaluated by a senior and junior radiologist, with more than 30 years of combined clinical experience. Technically, 3.5- to 5-mm-thick transversal and 5-mm-thick sagittal and coronal multiplanar-reformatted images were reviewed on a picture archiving and communication system (AGFA IMPAX; AGFA Health Care). Divergent findings were jointly assessed and the final results decided by consensus. Cases with non-existing trauma anamnesis (e.g. spontaneous splenic rupture) or where no lesion was found intraoperatively or on CT imaging re-evaluation were excluded. Previous external surgical or interventional procedures prior to initial radiographic assessment were also considered an exclusion criterion.

Splenic and hepatic lesions were classified according to the 1994-AAST classification [13, 14], the 2018-AAST classification [24], the CTSI for splenic injuries [21] and to a novel own adaptation for hepatic injuries of the previously published splenic CTSI (Table 2). Analogously, the hepatic CTSI scoring system describes four grades of injury, with grade IV being divided into two subgroups (IVa, IVb). According to the CTSI, liver injuries up to grade III are graded following the morphological criteria, in a similar way to the 1994-AAST classification [13, 14]. Considering the differences between spleen and liver dimensions, hepatic parenchymal injuries < 5 cm are classified as grade II. Parallel to the splenic CTSI, intraparenchymal, subcapsular or intraperitoneal contrast extravasations are graded as IVa/IVb, respectively. Hepatic vascular injuries (pseudoaneurysm or arteriovenous fistula) or completely shattered livers were considered grade IVa.

OM was defined as any abdominal surgical intervention during hospital stay, whereas NOM included interventional radiology (e.g. angiography, drainage), endoscopy (e.g. endoscopic retrograde cholangio-pancreaticography) and all non-interventional medical therapies. Indication for primary OM was suspicion of hollow-organ perforation or persistent hemodynamic instability despite appropriate emergency fluid resuscitation and coagulation management as previously described [26]. Indications for secondary OM were recurrent instability due to bleeding or abdominal septic complications.

Clinical data collected included patient age and sex, trauma cause, accompanying extra-abdominal injuries, Glasgow Coma Scale (GCS) score, initial management (NOM vs. OM), failure of NOM and rate of secondary OM, in-hospital mortality with cause of death and length of stay (LOS). The injury severity score (ISS) was calculated by the addition of each body regions’ abbreviated injury score (AIS) [27].

### Statistical analysis

Data are presented as numbers and proportions, continuous variables as mean with standard deviation (SD) or median with interquartile range (IQR). Differences between injury groups, radiological classification and treatment outcomes are calculated using *χ*^2^ or Fisher’s exact test (< 5 cases per group) for categorical variables and Kruskal-Wallis test for continuous variables; normal distribution was assessed by the Shapiro-Wilk test. The diagnostic ability of radiological classifications stratified by mild versus severe injury to predict mortality was assessed through diagnostic accuracy (proportion of severe cases with mortality and mild cases without mortality among all subjects), diagnostic odds ratio (DOR; odds of severe injury in deaths divided by severe injuries in survivors), positive predictive value (PPV; severe cases with mortality among severe cases) and negative predictive value (NPV; mild cases without mortality among mild cases). Correlation between grading and therapeutic management was calculated with Cramer’s V, with a level of > 0.250 indicating strong correlation. For all tests, *p* values < 0.05 were considered significant. SPSS 21.0 (IBM Corporation) and OpenEpi 3.01 (www.openepi.com) were used for analysis.

## Results

### Patients, management and outcomes

Between 2000 and 2016, in total, 731 patients with radiologically or intraoperatively confirmed blunt splenic or hepatic lesions were treated at our hospital. After the exclusion of patients with pre-admission external surgical or interventional treatment and cases with missing imaging files (*n* = 28), a total of 703 patients were included in the analysis.

Patient characteristics are displayed in Table 4. In summary, 271 cases presented with splenic injury, 352 with hepatic and 80 with hepatosplenic injury. The mean age was 32.9 years and 68.1% of patients were male; the main causes of trauma were winter sports (33%) and car (22%) and motorcycle accidents (12.1%). The majority (85.8%) of patients had polytrauma (ISS > 15). Cases with combined hepato-splenic injuries were significantly more severely injured compared with splenic or hepatic injuries in terms of GCS, ISS, associated extra-abdominal injuries (except facial injuries) and haemoglobin levels on admission (all *p* < 0.05). This also resulted in an increased LOS and in-hospital mortality in the hepato-splenic injury subgroup. The overall mortality was 4.8%, most commonly due to intracranial hypertension (32.4%) and sepsis (20.6%).

**Table 4:** Patient characteristics and injury details

	All patients, *n* = 703 (%)	Splenic injury, *n* = 271 (%)	Hepatic injury, *n* = 352 (%)	Combined splenic and hepatic injury, *n* = 80 (%)	*p* value*
Male	479 (68.1)	211 (77.9)	204 (58)	64 (80)	< 0.001
Age (mean; SD)	32.9 (18.0)	32.6 (18.0)	33.7 (18.1)	30.7 (17.3)	0.321**
Trauma cause (missing = 2)					0.017
Car accident	154 (22.0)	58 (21.4)	71 (20.3)	25 (31.3)	
Motorcycle accident	85 (12.1)	34 (12.5)	39 (11.1)	12 (15)	
Pedestrian or comparable occupational accidents	33 (4.7)	8 (3.0)	20 (5.7)	5 (6.3)	
Cycling accident	57 (8.1)	19 (7.0)	36 (10.3)	2 (2.5)	
Winter sports	231 (33.0)	99 (36.5)	116 (33.1)	16 (20)	
Fall from heights	74 (10.6)	25 (9.2)	36 (10.3)	13 (16.3)	
Minimal trauma (e.g. in homely environment)	45 (6.4)	24 (8.9)	17 (4.9)	4 (5)	
Personal assault	2 (0.3)	1 (0.4)	1 (0.3)	0 (0)	
Horse riding accident (or other animal associated injuries)	20 (2.9)	3 (1.1)	14 (4.0)	3 (3.8)	
GCS-Score on admission (missing = 15): mean (SD)	13.0 (3.6)	13.2 (3.4)	13.1 (3.5)	11.6 (4.3)	< 0.001**
GCS ≤ 8 (unconsciousness)	105 (15.2)	32 (12.1)	51 (14.7)	22 (27.8)	0.003
Injury severity score (ISS): median (SD)	27.0 (12.7)	25.0 (12.6)	27.0 (11.6)	34.0 (14.3)	< 0.001**
ISS > 15 (definition of polytrauma)	603 (85.8)	197 (72.7)	330 (93.8)	76 (95)	< 0.001
Associated extra-abdominal injuries (AIS score ≥ 1)					
Head or neck	263 (37.4)	84 (31)	140 (39.8)	39 (48.8)	0.007
Face	99 (14.1)	31 (11.4)	56 (15.9)	12 (15)	0.274
Chest	452 (64.3)	166 (61.3)	225 (63.9)	61 (76.3)	0.048
Extremities or pelvic girdle	335 (47.7)	120 (44.3)	166 (47.2)	49 (61.3)	0.027
External (skin and soft tissue)	443 (63)	149 (55)	229 (65.1)	65 (81.3)	< 0.001
Haemoglobin on admission (missing = 16): mean (SD)	115.7 (24.6)	117.7 (25.6)	116.5 23.4)	105.0 (23.6)	< 0.001**
Thrombocytes on admission (missing = 16): mean (SD)	193.8 (70.9)	192.6 (74.6)	197.0 (67.9)	183.7 (70.6)	0.113**
Length of hospital stay (days) (missing = 4): Median (IQR)	14 (13)	13 (14)	13 (12)	19 (16)	0.004**
Mortality (in-hospital)	34 (4.8)	10 (3.7)	16 (4.5)	8 (10)	0.065
Cause of death (% of deaths)					0.236
Sepsis	7 (20.6)	3 (30)	4 (25)	0 (0)	
Haemorrhagic shock	3 (8.8)	0 (0)	2 (12.5)	1 (12.5)	
Intracranial hypertension	11 (32.4)	2 (20)	7 (43.8)	2 (25)	
Multiorgan failure	5 (14.7)	1 (10)	2 (12.5)	2 (25)	
Cardiac dysfunction/infarction	1 (2.9)	0 (0)	0 (0)	1 (12.5)	
Arrived with CPR/no ROSC	5 (14.7)	2 (20)	1 (6.3)	2 (25)	
Other/unknown	2 (5.9)	2 (20)	0 (0)	0 (0)	

*All *p* values were calculated with chi-square-test or Fisher’s exact test except for ** (Kruskal-Wallis test). *GCS*, Glasgow Coma Scale; *ISS*, injury severity score; *AIS*, associated injury score; *IQR*, interquartile range; *SD*, standard deviation; *CPR*, cardio-pulmonary resuscitation; *ROSC*, return of spontaneous circulation

Primary NOM was applied in 71.6% of patients with splenic trauma, 94% of patients with hepatic and 73.8% with hepato-splenic injury. Although NOM was successful in over 90% of cases in all subcategories, it was significantly more often leading to secondary OM in splenic injuries (5.7%) and hepato-splenic injuries (5.1%), than in patients with liver injury only (1.8%; *p* = 0.037). In-hospital mortality was 10.9% in primary OM patients and 3.6% in NOM patients (*p* = 0.001). In OM cases, the mortality was 6.5% for splenic, 14.3% for liver and 23.8% for combined hepato-splenic injury (*p* = 0.054). In NOM cases, in-hospital mortality was 2.6% for splenic, 3.9% for liver and 5.1% for hepato-splenic injury (*p* = 0.503).

### Radiological injury severity grading

Radiological grading according to different classifications and injury sub-groups is represented in Table 5. To facilitate comparison of the AAST classifications and CTSI, grade I–III injuries were considered “mild”, whereas all injuries > grade III were classified as “severe”. When graded according to the 1994-AAST classification, severe injuries were found in 24% of patients with splenic injury, 19.9% with hepatic injury and 30.0% with hepato-splenic injury, whereas according to the 2018-AAST revision, severe injuries were recorded in 35.1%, 20.2% and 36.3%, respectively. Classified according to CTSI, 26.9% of patients with splenic, 7.1% with hepatic and 23.8% with hepato-splenic trauma had severe injuries.

**Table 5:** Radiological findings according admission CT scan stratified by injured organ

	All patients *n* = 703 (%)	Splenic injury *n* = 271 (%)	Hepatic injury *n* = 352 (%)	Combined splenic and hepatic injury *n* = 80 (%)	Difference between groups (*p* value)*
AAST (Moore) injury score 1994					
Spleen					< 0.001
0	354 (50.4)	0 (0)	352 (100)	2 (2.5)^#^	
1	38 (5.4)	20 (7.4)	–	18 (22.5)	
2	65 (9.2)	44 (16.2)	–	21 (26.3)	
3	170 (24.2)	142 (52.4)	–	28 (35)	
4	47 (6.7)	41 (15.1)	–	6 (7.5)	
5	29 (4.1)	24 (8.9)	–	5 (6.3)	
Liver					< 0.001
0	271 (38.5)	271 (100)	0 (0)	0 (0)	
1	42 (6.0)	–	33 (9.4)	9 (11.3)	
2	91 (12.9)	–	72 (20.5)	19 (23.8)	
3	215 (30.6)	–	177 (50.3)	38 (47.5)	
4	66 (9.4)	–	57 (16.2)	9 (11.3)	
5	18 (2.6)	–	13 (3.7)	5 (6.3)	
AAST (Kozar) injury score 2018					
Spleen					< 0.001
0	354 (50.4)	0 (0)	352 (100)	2 (2.5)^#^	
1	38 (5.4)	20 (7.4)	–	18 (22.5)	
2	62 (8.8)	43 (15.9)	–	19 (23.8)	
3	137 (19.5)	113 (41.7)	–	24 (30.0)	
4	58 (8.3)	50 (18.5)	–	8 (10.0)	
5	54 (7.7)	45 (16.6)	–	9 (11.3)	
Liver					< 0.001
0	271 (38.5)	271 (100)	0 (0)	0 (0)	
1	42 (6.0)	–	33 (9.4)	9 (11.3)	
2	91 (12.9)	–	72 (20.5)	19 (23.8)	
3	210 (29.9)	–	176 (50.0)	34 (42.5)	
4	74 (10.5)	–	60 (17.0)	14 (17.5)	
5	15 (2.1)	–	11 (3.1)	4 (5.0)	
CT severity index (CTSI)					
Spleen					< 0.001
0	354 (50.4)	0 (0)	352 (100)	2 (2.5)^#^	
1	39 (5.5)	23 (8.5)	–	16 (20)	
2	77 (11.0)	54 (19.9)	–	23 (28.8)	
3	146 (20.8)	121 (44.6)	–	25 (31.3)	
4a	53 (7.5)	45 (16.6)	–	8 (10)	
4b	34 (4.8)	28 (10.3)	–	6 (7.5)	
Liver					< 0.001
0	271 (38.5)	271 (100)	0 (0)	0 (0)	
1	32 (4.6)	–	24 (6.8)	8 (10)	
2	138 (19.6)	–	109 (31)	29 (36.3)	
3	229 (32.6)	–	194 (55.1)	35 (43.8)	
4a	22 (3.1)	–	20 (5.7)	2 (2.5)	
4b	11 (1.6)	–	5 (1.4)	6 (7.5)	

*All *p* values were calculated with chi-square-test or Fisher’s exact test. *AAST*, American Association for Surgery of Trauma; ^#^In two patients, no splenic/hepatic injury was visible on CT but was detected intraoperatively in explorative laparotomy

**Fig. 1:**
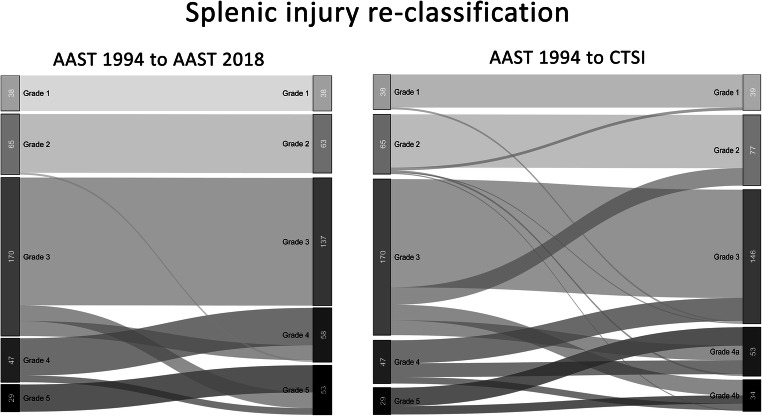
Re-classification according to the 2018-AAST classification and the CTSI compared with the 1994-AAST grading for splenic injury severity

**Fig. 2:**
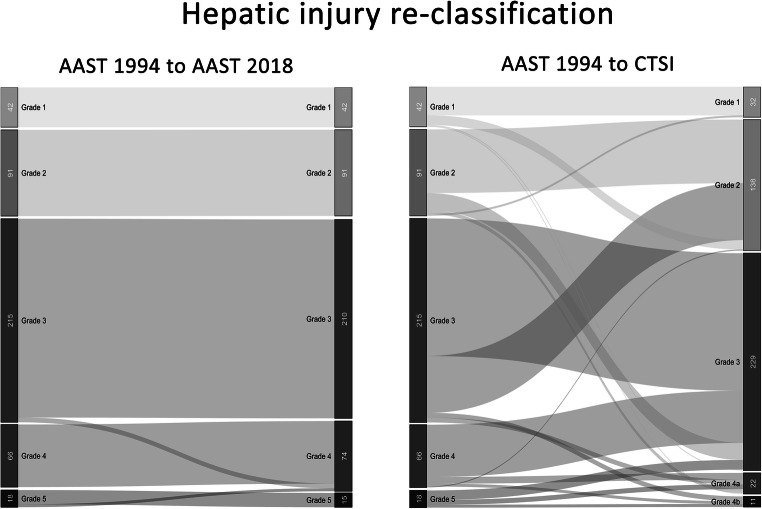
Re-classification according to the 2018-AAST classification and the CTSI compared with the 1994-AAST grading for hepatic injury severity

Figures 1 and 2 present grading re-arrangement when classifying patients according to the 2018-AAST and the CTSI compared with the 1994-AAST. In splenic trauma, re-classification to 2018-AAST resulted in changes of the individual injury severity in 98 cases (28.1%) including 36 mild injuries being upstaged to severe grades, while CTSI scoring changed severity in 122 cases (40.0%) with 36 mild injuries being upstaged to severe grades and 25 severe cases downstaged to mild grades. In liver trauma, re-classification to 2018-AAST resulted in changes in 8 cases (1.9%) including upstaging of 5 mild injuries, while CTSI scoring changed grading in 180 cases (41.7%) with 15 mild injuries being upstaged and 65 severe cases being downstaged.

### Prediction of outcome and correlation with therapeutic management

**Fig. 3:**
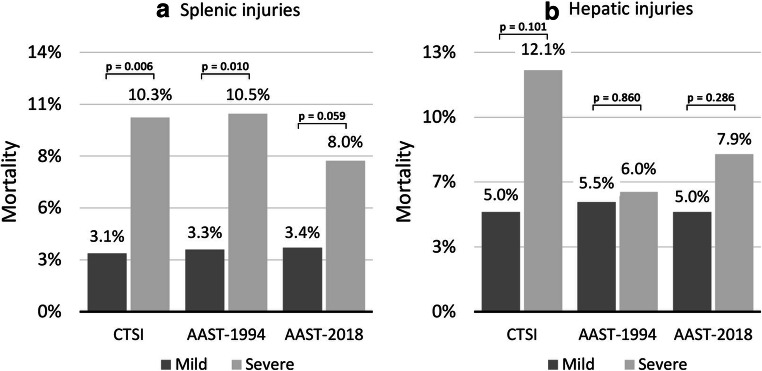
In-hospital mortality according to severity graded by CTSI, 1994-AAST and 2018-AAST in splenic injuries (**a**) and hepatic injuries (**b**). *P* values were calculated with chi-square test or Fisher’s exact test. CTSI, CT severity index; AAST-1994/2018, American Association for Surgery of Trauma 1994 and 2018 classification for splenic and hepatic injuries

As depicted in Fig. 3, severe splenic injuries according to all classifications were associated with a higher in-hospital mortality compared with mild injuries. The CTSI showed a diagnostic accuracy to predict mortality of 75.4% (95%CI 70.6–80.0, DOR 3.66 (95%CI 1.37–9.82), PPV, 10.3%; NPV, 97.0%; chi^2^ *p* = 0.006), which was comparable with the 1994-AAST (diagnostic accuracy 77.9% (95%CI 73.3–82.0), DOR 3.45 (95%CI 1.28–9.28), PPV 10.5%, NPV 96.7%; chi^2^ *p* = 0.010). In this regard, both were more accurate than the 2018-AAST revision (diagnostic accuracy 68.2% (95%CI 63.1–72.9), DOR 2.50 (95%CI 0.94–6.67), PPV 8.0%, NPV 96.6%; chi^2^ *p* = 0.059). In hepatic injuries, the CTSI discriminated better in terms of mortality prediction (diagnostic accuracy 88.7% (95%CI 85.3–91.3), DOR 2.61 (95%CI 0.84–8.16), PPV 12.1%, NPV 95%; chi^2^ *p* = 0.101), compared with the 2018-AAST classification (diagnostic accuracy 77.1% (95%CI 72.9–80.8), DOR 1.64 (95%CI 0.66–4.08), PPV 7.9%, NPV 95%; chi^2^ *p* = 0.286). The predictive accuracy of the 1994-AAST grading was limited, with no clinically relevant discrimination between mortality rates of mild (5.5%) and severe (6.0%) hepatic injury cases (diagnostic accuracy 77.3% (95%CI 73.1–81.0), DOR 1.10 (95% 0.40–3.03), PPV 6%, NPV 94.5%; chi^2^ *p* = 0.860).

**Fig. 4:**
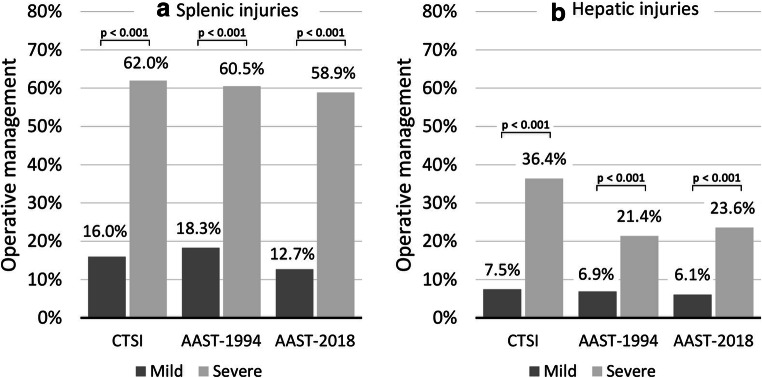
Primary operative management rates according to severity graded by CTSI, 1994-AAST and 2018-AAST in splenic injuries (**a**) and hepatic injuries (**b**). *P* values were calculated with chi-square test or Fisher’s exact test. CTSI, CT severity index; AAST-1994/2018, American Association for Surgery of Trauma 1994 and 2018 classification for splenic and hepatic injuries

Focusing on the type of primary treatment, in patients with splenic involvement (Fig. 4a), all three classification systems showed a highly significant association with the need for initial operative management (all *p* < 0.001)*.* However, the CTSI and the 2018-AAST classification showed a stronger correlation with decision for OM (Cramer V = 0.446 and 0.484) compared to the 1994-AAST classification (Cramer V = 0.390), better discriminating between mild injuries (16% and 12.7% OM, respectively) and severe injuries (62% and 58.9% OM, respectively) compared with the 1994-AAST classification (18.3% vs. 60.5%, respectively). The results calculated for hepatic injuries (Fig. 4b) furthermore showed a stronger correlation of the CTSI with the need for OM (mild vs. severe, 7.5% vs. 36.4%; Cramer V = 0.259; *p* < 0.001) compared with the 1994-AAST (mild vs. severe, 6.9% vs. 21.4%; Cramer V = 0.194; *p* < 0.001) and the 2018-AAST grading (mild vs. severe, 6.1% vs. 23.6%; Cramer V = 0.239; *p* < 0.001).

Finally, a composite endpoint of either primary need for OM or failure after primary NOM (secondary OM) was analysed. The CTSI and 2018-AAST were superior to the 1994-AAST classification in terms of correlation of severe injuries with the need for any operative treatment during the whole hospital stay in both splenic as well as hepatic injury patients (all *p* < 0.001). Classified according to CTSI, in spleen trauma, 69% of severe cases compared with 19.1% of mild cases underwent OM (Cramer V = 0.464) at any time point, compared with 65.2% and 15.6% according to the 2018-AAST classification (Cramer V = 0.498). In contrast, classified by 1994-AAST, 65.8% of severe and 22% of mild cases (Cramer V = 0.389) needed OM. Regarding liver trauma, 42.4% of severe and 9.3% of mild CTSI patients (Cramer V = 0.273) compared with 30.3% of severe and 7.0% of mild 2018-AAST cases (Cramer V = 0.293) and 28.6% of severe and 7.8% of mild 1994-AAST patients (Cramer V = 0.255) in total underwent OM.

## Discussion

Our study included > 700 patients with blunt splenic and hepatic injury, treated at a central European trauma unit. With more than 85% of cases classified as polytraumatic, 83% treated primarily with NOM and an overall mortality < 5%, this cohort represents a solid basis for radiological evaluation within a state-of-the-art environment. Re-evaluation of CT images confirmed that the splenic CTSI incorporating contrast media extravasation, as previously proposed [21], is comparable with the 1994-AAST classification in terms of accuracy to predict mortality but is correlating better with the need for primary or secondary OM. With the evaluation of a modified liver injury version of the CTSI, we furthermore showed that this CT score facilitates enhanced outcome prediction and management guidance in hepatic injuries. Finally—although in a slightly different way compared with the CTSI—the 2018-AAST classifications for splenic and hepatic trauma also incorporate contrast extravasations to better account for vascular injury features [24]. The present study representing the first to validate this update in an independent cohort shows that the revised 2018 version was superior to the 1994-AAST classification in terms of correlation with necessity for primary or secondary OM with comparable significance as the CTSI. However, it was clearly inferior to the CTSI for the prediction of mortality (Figs. 3 and 4).

We also analysed the differences between the 2018-AAST and CTSI in terms of re-grading patients compared with the 1994-AAST (Figs. 1 and 2). Especially in cases with hepatic injury, the 2018-AAST classification fails to provide more accuracy in terms of mortality prediction, which is mainly caused by patients being classified as severe due to large parenchymal defects without contrast extravasation. Results of the CTSI grading analysis suggest that primarily the presence of intraperitoneal extravasation on CT constitutes the most critical factor for mortality in blunt trauma patients.

Other studies [19–21, 23, 28] have previously shown that the presence of splenic vascular injuries is a predictor of the need for OM. More than 20 years ago, Schurr et al already suggested that an intraparenchymal contrast blush on CT was an important management consideration [20]. More recently, Saksobhavivat et al have analysed the CTSI in 171 patients with splenic injury suggesting that this score represented a substantially better individual predictor of a successful observation than other factors like abdominal AIS, hemoperitoneum volume or different vital and laboratory parameters on admission [23]. In contrast to our study, they did not evaluate the value of the CTSI to predict mortality.

Regarding blunt liver injury, we present the first evidence evaluating an adapted version of the CTSI. Concerning hepatic contrast extravasation in general, a previous study from Taiwan [29] conducted in the year 2000 examined the exact location of a CT contrast blush. Although with limited study power, they have shown that all patients with intraperitoneal contrast media pooling became hemodynamically unstable early after admission and required laparotomy, compared with 67% of cases with mixed intraparenchymal intraperitoneal bleeding and 0% with isolated intraparenchymal extravasation. Although the utilisation rate of NOM has increased since even in high-grade injuries, their findings are still in concordance with our data, where 23% of CTSI IVa (bleeding within the liver) patients compared with 82% CTSI IVb (intraperitoneal bleeding) cases ultimately required OM at some time during the hospital stay (*p* = 0.002). The findings in our splenic injury patients are less pronounced with 62% of patients with CTSI IVa requiring OM compared with 79% with CTSI IVb (*p* = 0.092). In summary, consistent with previous results, the presence and location of active extravasation on MDCT in our series correlated with outcome and necessity of OM management in both splenic as well as hepatic trauma patients [19, 21].

Our study had several limitations, primarily owed to its retrospective, single-centre design. At our hospital, all polytraumatic patients are evaluated by an interdisciplinary team and initial management is determined on consensus, depending on injury severity and comorbidities with no standardised internal algorithm currently in place. Another limitation was the variability of MDCT protocols on admission imaging. In all cases, portal venous phase contrast enhancement abdominal MDCTs with multiplanar reformation were performed. Selectively, arterial or LATE venous phase enhancement imaging was obtained to gather additional information. A previous study showed that routine addition of an arterial phase might further increase sensitivity in detecting active haemorrhage [30]. Also, interobserver and intraobserver variabilities of the CTSI/AAST were not determined in our study. However, these were considered almost perfect for the splenic CTSI in a previous Dutch study with kappa values > 0.8 [31]. In our experience, compared with the AAST classifications, the CTSI proved easier application because it uses the same measurements for all or low-grade injury types and it is usually faster to detect contrast extravasation than to speculate with percental parenchymal involvement of high-grade injuries. Furthermore, as previously suggested, implementing a clinical algorithm for routine angiography in high-grade patients could potentially further decrease the rate of OM especially in splenic injury patients and improve outcomes [26, 32]. Lastly, this cohort represents a typical trauma population of a Central European alpine centre with many male winter sports patients and decreasing traffic accident injuries over the years [26]. Therefore, our findings may not directly translate to other areas with different trauma mechanisms.

In conclusion, the implementation of contrast media extravasation into injury severity gradings as proposed by both the CTSI and the 2018-AAST classifications shows substantial advantages over the previous 1994-AAST staging in regard to prediction of a necessity for operative management in blunt splenic and hepatic injury patients. However, the CTSI proved to better predict in-hospital mortality compared with the 2018-AAST classification. Because of its easy, fast and reproducible application, the CTSI might currently substitute the AAST-OIS as the gold standard screening tool in early management decision-making processes of blunt liver and spleen trauma. Our data suggest that further revalidation and fine-tuning of the 2018-AAST classification seems advisable to facilitate clinical applicability.

## Abbreviations

AAST-OIS: American Association of Surgery of Trauma Organ Injury Scale
AIS: Abbreviated injury score
CPR: Cardio-pulmonary resuscitation
CTSI: CT severity index
GCS: Glasgow Coma Scale
IQR: Interquartile range
ISS: Injury severity score
LOS: Length of stay
NOM: Non-operative management
OM: Operative management
ROSC: Return of spontaneous circulation
SD: Standard deviation
